# Core–Shell Composite GaP Nanoparticles with Efficient Electroluminescent Properties

**DOI:** 10.3390/ma18030487

**Published:** 2025-01-22

**Authors:** Duo Chen, Ruiyuan Bi, Lifeng Xun, Xiaoyan Li, Qingyu Hai, Yao Qi, Xiaopeng Zhao

**Affiliations:** Smart Materials Laboratory, Department of Applied Physics, Northwestern Polytechnical University, Xi’an 710129, China; chenduo@mail.nwpu.edu.cn (D.C.); biruiyuan@mail.nwpu.edu.cn (R.B.); xunlifeng@mail.nwpu.edu.cn (L.X.); lixiaoyan0521@mail.nwpu.edu.cn (X.L.); haiqingyu@mail.nwpu.edu.cn (Q.H.); qiyao@mail.nwpu.edu.cn (Y.Q.)

**Keywords:** thermal injection method, core–shell composite structure, GaP nanoparticles, electroluminescence

## Abstract

Gallium-based light-emitting diodes (LEDs), including AlGaInP and GaN, have become the most widely used light-emitting devices in modern scientific research and practical applications. However, structures like carrier injection layers, active layers, and quantum well layers ensure the high luminescence efficiency of LEDs but also limit their applications at the micro- and nanoscale. Although the next generation of micrometer-scale light-emitting diodes (Micro-LEDs) has alleviated these issues to some extent, challenges such as edge effects and etching damage caused by size reduction lead to lower luminous efficiency and shorter lifetimes. Inspired by LED structure, this study designed and synthesized core–shell composite GaP:Zn/GaP/GaInP and GaP:Te/GaP nanoparticles using a thermal injection method. After high-temperature annealing, these composite materials demonstrated efficient electroluminescent performance under electric field excitation through band-edge transitions and the Zn_Ga_-O_P_ recombination mechanism. Experimental results show that the GaP:Zn/GaP/GaInP-GaP:Te/GaP composite samples with doping concentrations of 15%Zn-8%Te, a core–shell precursor ratio of 1:1:1, and reaction times of 1 h:20 min:20 min exhibit the best electron–hole injection efficiency and bound-recombination efficiency. Under excitation by an external electric field, they demonstrated optimal electroluminescence performance, with a relative luminous intensity of 11,109.21 at 600 nm, approximately 15 times higher than that of the initial condition samples. In addition, this study systematically investigated the structure, morphology, and elemental composition of the composite materials using various characterization techniques, including X-ray diffraction (XRD), transmission electron microscopy (TEM), scanning electron microscopy (SEM), energy-dispersive X-ray spectroscopy (EDS), and X-ray photoelectron spectroscopy (XPS). These GaP-doped nanoparticles with a core–shell composite structure, inspired by LED design, exhibited outstanding electroluminescent performance, providing new insights into the development of novel micro- and nanoscale electroluminescent materials.

## 1. Introduction

Since Holonyak et al. invented the GaAsP red light-emitting diode (LED) in 1962, gallium-based semiconductors have gradually become the preferred materials for visible light LEDs, evolving into two major systems: GaN and AlGaInP [1]. GaN-LEDs emit visible light from violet to green, while AlGaInP-LEDs cover the yellow-green to red light regions [2,3,4,5]. Benefiting from their small size, low power consumption, high efficiency, long lifespan, and environmental advantages, LEDs have wide application prospects in fields such as lighting, displays, sensing, and healthcare [6,7,8,9]. Between 2021 and 2024, our group constructed smart meta-superconductors (SMSCs) by combining GaN and AlGaInP commercial LEDs with MgB_2_ and BSCCO superconductors. By leveraging the electroluminescent properties of LEDs, the superconductivity such as critical transition temperature, critical magnetic field and critical current of MgB_2_ and B(P)SCCO were significantly improved [10,11,12,13]. Although modern LEDs improve electron–hole recombination efficiency by incorporating functional layers such as active layers and quantum well layers (e.g., introducing InGaN layers in GaN-LEDs [14,15,16] and GaInP layers in AlGaInP [17,18]) to achieve excellent light emission performance, the electroluminescent intensity of LEDs significantly decreases at the micro/nanoscale, making it difficult to meet the demands for high-efficiency light sources in our superconducting project. Furthermore, LEDs still face limitations such as complex fabrication processes, high costs, and the toxicity of raw materials [19,20]. Therefore, it is imperative to develop a new type of efficient electroluminescent material with a micro or nano size.

Based on the current research status, this study aims to conduct related research starting from the GaP system. GaP is an environmentally friendly indirect bandgap semiconductor, and the involvement of phonons leads to a low probability of electronic transitions between energy levels, resulting in weak photoluminescence intensity after excitation. An effective solution is to use elemental doping to introduce spatially localized electronic defects in the GaP crystal, which extends the electron wavefunction in k-space and forms quasi-direct bandgap transitions [21]. Early researchers achieved electron wavefunction extension in momentum space by substituting N for P to form isoelectronic defects, effectively promoted the band-edge transitions of GaP. Building on this, doping Zn and Te into the GaP matrix to form individual P-type and N-type layers, and constructing a P–N structure allows for green luminescence based on the GaP bandgap when a forward bias is applied [22]. The partial substitution of O for P not only has an equivalent effect, but also forms a Zn_Ga_-O_P_ composite center with Zn_Ga_ within the diode, which produces red luminescence via donor–acceptor recombination under electric field excitation [23]. Additionally, the strong quantum confinement effects can also be utilized to mitigate the indirectness of the GaP energy band structure to enhance its optical activity [24,25,26], which places high demands on the synthesis of GaP nanoparticles. Early researchers attempted to synthesize GaP nanoparticles using methods such as dehalogenation, precursor decomposition, solvothermal synthesis, and ion exchange. However, these nanoparticles exhibited broad size distributions and failed to achieve breakthroughs in luminescent performance [27,28,29,30,31,32]. Subsequently, building on the well-established synthesis techniques for InP QDs, GaP nanoparticles with photoluminescent properties were synthesized using tris(trimethylsilyl)phosphine ((TMS)_3_P) as the phosphorus precursor via the thermal injection method. For example, Kim et al. synthesized GaP nanoparticles using GaCl_3_ and palmitic acid, achieving distinct blue emission at 485 nm with a quantum yield of approximately 15% [33]. Choi et al., using Ga(acac)_3_ and oleic acid, achieved a quantum yield as high as 40% for GaP nanoparticles, with effective size control and tunable emission ranging from 400 nm to 520 nm by adjusting the oleic acid content [34]. Beyond photoluminescence, Q-LEDs with GaP nanoparticles as the luminescent layer exhibit excellent luminescent performance under external electric field excitation. However, while Q-LEDs are typically prepared as thin films via spin-coating, the luminescence in their particle morphology is rarely reported [35,36,37].

In summary, in response to the limitations of current LED light-emitting materials and inspired by the electroluminescent principles of LEDs and the preparation process of GaP nanoparticles, this study synthesized Zn- and Te-doped GaP nanoparticles using the simple thermal injection method. With reference to the LED structure, GaP active layers and GaInP quantum well layers were coated on the outer surface of the doped GaP nanoparticles to achieve the effective electroluminescence in GaP:Zn/GaP/GaInP and GaP:Te/GaP particles. The morphology and structure of the GaP particles are characterized using techniques such as X-ray diffraction (XRD), transmission electron microscopy (TEM), scanning electron microscopy (SEM), energy-dispersive X-ray spectroscopy (EDS), and X-ray photoelectron spectroscopy (XPS). The effects of factors such as doping concentration, functional layer thickness, and reaction time on the electroluminescent performance of GaP:Zn/GaP/GaInP and GaP:Te/GaP particles are systematically studied to optimize light emission performance.

## 2. Materials and Methods

### 2.1. Materials

All the experimental materials used in this study are listed in Table 1.

### 2.2. Preparation of Samples

The preparation processes for GaP:Zn/GaP/GaInP and GaP:Te/GaP are introduced as examples. The samples were synthesized using a doping concentration of 15%Zn and 8%Te, a core–shell precursor ratio of 1:1:1, and a reaction time of 1 h:20 min:20 min.

Synthesis of GaP:Zn:

In a 10 mL three-neck flask, 0.25 mmol of Ga(acac)_3_, 0.0375 mmol of Zn(acac)_3_, 0.125 mmol of OA, and 5 mL of ODE were added. The reaction system was purged with Ar at 130 °C for 1.5 h to expel air. The temperature was then raised to 300 °C, and a mixture of 0.125 mmol (TMS)_3_P and 0.5 mmol TOP was swiftly injected. This caused the temperature of the reaction system to drop instantly to approximately 280 °C. The solution was then reheated to 300 °C and maintained under Ar for 1 h. The resulting colloidal solution was cooled to room temperature under an Ar atmosphere. The reaction product was washed with ethanol and centrifuged at 9000 rpm for 15 min to precipitate the solids, discarding the supernatant. This washing process was repeated three to four times. The resulting precipitate was dissolved in 1.5 mL of n-hexane, forming the GaP:Zn-hexane solution.

Synthesis of GaP:Te:

The synthesis process for GaP:Te is similar to that for GaP:Zn. In a 10 mL three-neck flask, 0.25 mmol of Ga(acac)_3_, 0.125 mmol of OA, and 5 mL of ODE were added. The mixture was purged with Ar at 130 °C for 1.5 h to remove air. The temperature was then raised to 300 °C, and a mixture of 0.125 mmol (TMS)_3_P, 0.01 mmol of Te powder, and 0.5 mmol TOP was swiftly injected, followed by a 1 h reaction. The mixture was then cooled to room temperature, and the reaction product was washed with ethanol. The resulting precipitate was dissolved in 1.5 mL of n-hexane, forming the GaP:Te-hexane solution.

Synthesis of GaP Active Layer:

A single GaP active layer was grown on the GaP:Zn (or GaP:Te) core using a similar process. Specifically, 0.25 mmol of Ga(acac)_3_, 0.125 mmol of OA, 5 mL of ODE, and the GaP:Zn-hexane solution (or GaP:Te-hexane solution) were added to a 10 mL three-neck flask. The mixture was heated to 130 °C and purged with Ar for 1.5 h to remove air and n-hexane. The temperature was then raised to 300 °C, and a mixture of 0.125 mmol (TMS)_3_P and 0.5 mmol TOP was swiftly injected and reacted for 20 min. The reaction product was washed with ethanol, and the resulting precipitate was dissolved in 1.5 mL of n-hexane, forming the GaP:Zn/GaP-hexane solution (or GaP:Te/GaP-hexane solution).

Synthesis of GaInP Quantum Well Layer:

A single layer of GaInP quantum well was grown on the GaP:Zn/GaP core. In a 10 mL three-neck flask, 0.25 mmol of Ga(acac)_3_, 0.025 mmol of InCl_3_, 0.125 mmol of OA, 5 mL of ODE, and the GaP:Zn/GaP-hexane solution were added. The mixture was heated to 130 °C and purged with Ar for 1.5 h to remove air and n-hexane. The temperature was then raised to 300 °C, and a mixture of 0.125 mmol (TMS)_3_P and 0.5 mmol TOP was swiftly injected and reacted for 20 min. The reaction product was washed with ethanol, and the resulting precipitate was dissolved in 1.5 mL of n-hexane, forming the GaP:Zn/GaP/GaInP-hexane solution.

### 2.3. Characterizations

XRD data of the samples were analyzed using a Brucker D8 Advance X-ray diffractometer (Billerica, MA, USA) (Cu Kα radiation, wavelength 0.15406 nm, scanning step 0.02°, scanning speed 12.5 s/°). The surface morphology, elemental composition, and distribution of the samples were analyzed using an FEI Verios G4 (Hillsboro, OR, USA) field emission scanning electron microscope (SEM), an FEI Talos F200 (Hillsboro, OR, USA) transmission electron microscope (TEM), and a ThermoFisher Thermo NS7 (Waltham, MA, USA) energy dispersive X-ray spectrometer (EDS). X-ray photoelectron spectroscopy (XPS) data were obtained using a Thermo Scientific K-Alpha XPS spectrometer (Waltham, MA, USA), with the C 1s peak at 284.8 eV used as a reference to calibrate the elemental binding energies. The electroluminescence (EL) spectra of the samples were measured using an Ocean Optics USB2000 fiber optic spectrometer (Orlando, FL, USA).

Sample pretreatment is required for testing the electroluminescence properties. Equal amounts of GaP:Zn/GaP/GaInP-hexane solution and GaP:Te/GaP-hexane solution were mixed in a sample vial, followed by 0.5 h of ultrasonic vibration and 0.5 h of stirring to ensure thorough mixing. The mixed solution was evenly applied to Al_2_O_3_ substrates, dried at 150 °C, and then annealed at high temperature in an Ar atmosphere. The temperature control program for annealing was set as follows: starting at room temperature, heating at 5 °C/min to 300 °C, followed by heating at 10 °C/min to 860 °C, holding for 10 min, and finally cooling at 10 °C/min to room temperature. The annealed products were milled for 1 h and tested for their electroluminescence properties.

## 3. Results and Discussion

### 3.1. Phase Identification

**Figure 1:**
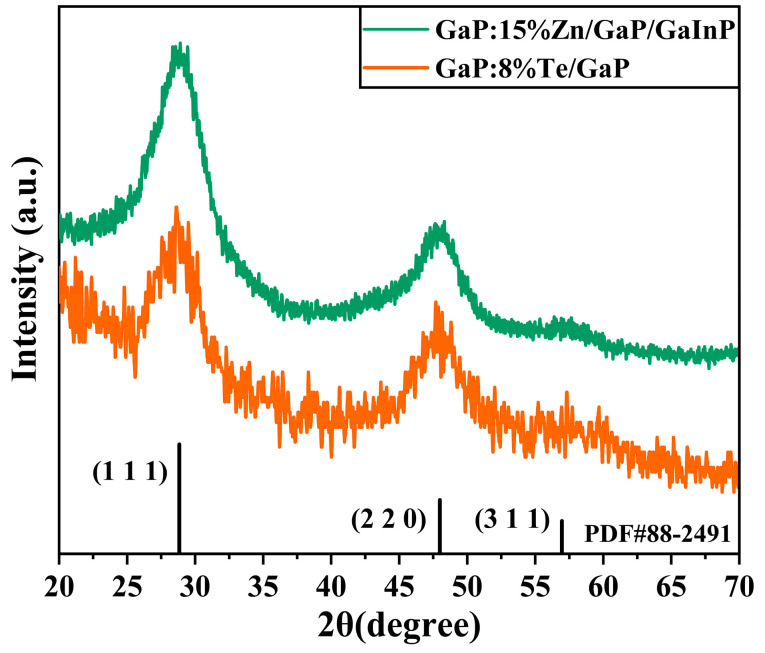
XRD patterns of GaP:15%Zn/GaP/GaInP and GaP:8%Te/GaP samples.

The crystal structure of the samples was first characterized using XRD. Figure 1 shows the XRD patterns of the GaP:Zn/GaP/GaInP and GaP:Te/GaP samples synthesized using the thermal injection method with a doping concentration of 15%Zn-8%Te. The diffraction peaks of both samples match perfectly with the zinc blende structure of GaP, pointing to the (111), (220), and (311) crystal planes, respectively, which are in agreement with the standard PDF#88-2491 and consistent with the results in the relevant literature [33,34]. No diffraction peaks for impurities were detected in the XRD patterns, indicating that Zn, Te, and In ions were successfully doped into the GaP crystal structure without forming elemental segregation or other impurity phases. The XRD peaks of GaP:Zn/GaP/GaInP and GaP:Te/GaP show no significant shifts, demonstrating that the low-concentration doping of Zn, In, and Te ions did not induce detectable lattice distortions in the GaP lattice [38,39,40]. Additionally, the GaP nanoparticles synthesized by the thermal injection method exhibit broad full-width at half maximum (FWHM), low diffraction intensity, and low signal-to-noise ratios, reflecting that the GaP nanoparticles have insufficient crystallinity and relatively small particle sizes.

### 3.2. Surface Morphology

**Figure 2:**
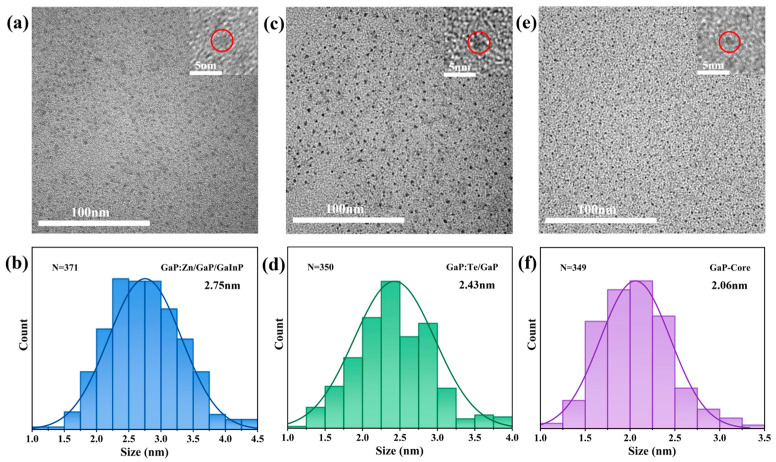
TEM images and particle size distribution of GaP nanoparticles (The red circle highlights a single GaP nanoparticle). (**a**,**b**) GaP:Zn/GaP/GaInP. (**c**,**d**) GaP:Te/GaP. (**e**,**f**) Pure GaP core.

The structural characteristics of materials directly influence their physical and chemical properties. TEM was utilized to examine the surface morphology of the samples. Figure 2a,c show the TEM images of GaP:Zn/GaP/GaInP and GaP:Te/GaP samples (doping concentration: 15%Zn-8%Te, core–shell reaction time: 1 h:20 min:20 min, precursor ratio: 1:1:1) taken at the same magnification, with insets displaying the magnified images of individual particles. Both samples exhibit the typical tetrahedral morphology of GaP nanoparticles. However, due to the presence of OA ligands on the surface, the imaging of the nanoparticles is slightly blurred. Based on the TEM images, the particle size distribution of approximately 350 GaP:Zn/GaP/GaInP and GaP:Te/GaP nanoparticles was analyzed, as shown in Figure 2b,d. The results show that the particle sizes of the samples follow a normal distribution, with average diameters of 2.75 nm for GaP:Zn/GaP/GaInP and 2.43 nm for GaP:Te/GaP. To verify that core–shell GaP nanoparticles were synthesized, we prepared pure GaP nanoparticle cores using the same experimental procedure and reaction parameters. The morphology and particle size distribution of the pure GaP cores are shown in Figure 2e,f. The average size of the pure GaP cores is 2.06 nm, which indicates that double-shell GaP:Zn/GaP/GaInP and single-shell GaP:Te/GaP nanoparticles with a core–shell structure were successfully synthesized by the thermal injection method. 

**Figure 3:**
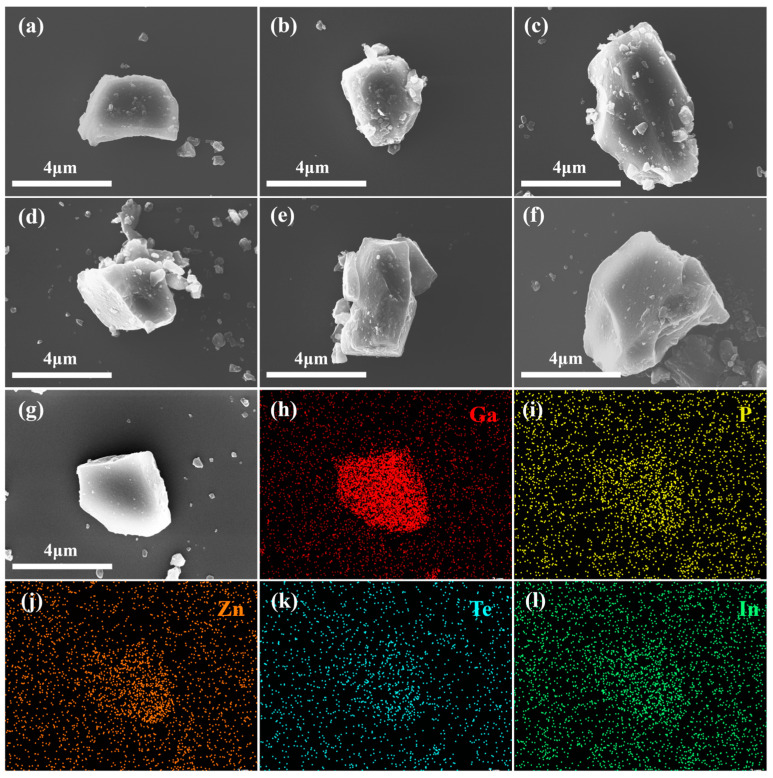
SEM images and EDS spectra of GaP:Zn/GaP/GaInP and GaP:Te/GaP after mixing, annealing, and grinding (doping concentration: 15%Zn-8%Te, core–shell reaction time: 1 h:20 min:20 min, precursor ratio: 1:1:1). (**a**–**g**) SEM images of individual particles. (**h**–**l**) Ga, P, Zn, Te, and In element mapping of the particles shown in (**g**).

In order to perform electroluminescence performance tests, two types of nanoparticles, GaP:Zn/GaP/GaInP and GaP:Te/GaP, were homogeneously mixed, annealed at high temperature, and ground. We then examined the surface morphology of the particles after mixing, annealing, and grinding using a scanning electron microscope (SEM), and acquired a 2D elemental mapping image of one of the particles. As shown in Figure 3, high-temperature annealing caused severe agglomeration of the samples. After grinding, the particles appeared irregular in shape, with an average size of 3–5 μm. In addition, two-dimensional EDS analysis of the annealed and ground GaP particles showed homogeneous distribution of Zn, In, and Te elements in the particles. This indicates that after ultrasonic vibration and stirring, the GaP:Zn/GaP/GaInP and GaP:Te/GaP nanoparticles were uniformly mixed, laying the foundation for their efficient electroluminescence performance.

### 3.3. Element Composition

**Figure 4:**
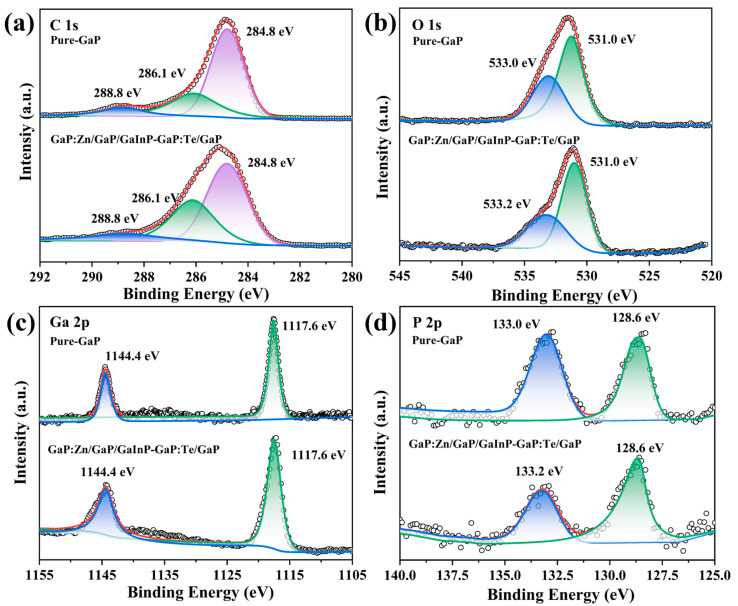
XPS high-resolution spectra of the matrix elements in GaP:Zn/GaP/GaInP-GaP:Te/GaP (doping concentration: 15%Zn-8%Te, core–shell reaction time: 1 h:20 min:20 min, precursor ratio: 1:1:1) and pure GaP samples. (**a**) C 1s. (**b**) O 1s. (**c**) Ga 2p. (**d**) P 2p.

**Figure 5:**
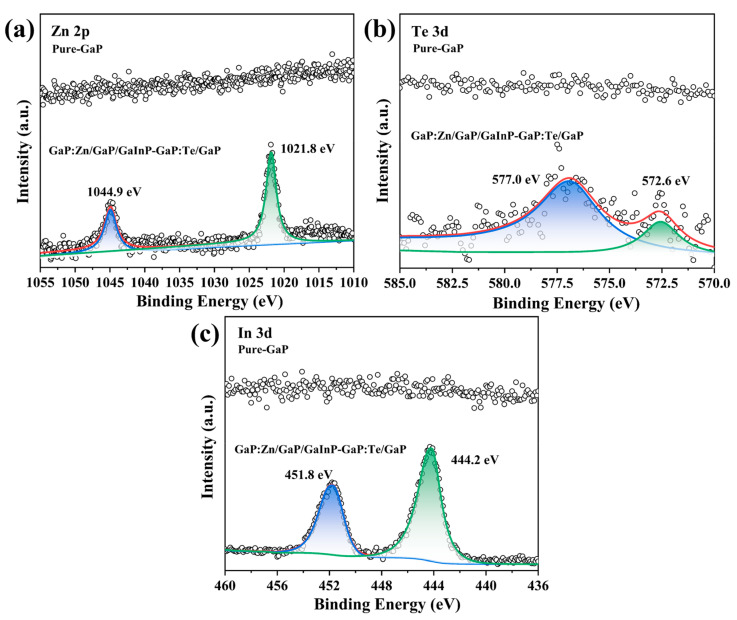
XPS high-resolution spectra of the doped elements in GaP:Zn/GaP/GaInP-GaP:Te/GaP (doping concentration: 15%Zn-8%Te, core–shell reaction time: 1 h:20 min:20 min, precursor ratio: 1:1:1) and pure GaP samples. (**a**) Zn 2p. (**b**) Te 3d. (**c**) In 3d.

The elemental composition and valence information of multilayered core–shell GaP nanoparticles were further analyzed and explored using XPS, a surface-sensitive materials testing technique that can typically only detect atoms within approximately 10 nm of the sample surface. Since the experimentally prepared nanoparticles are only a few nanometers in size, XPS is well-suited to analyze the elemental composition of the entire GaP nanoparticle. Figure 4 and Figure 5 show the high-resolution spectra of C, O, Ga, P, Zn, Te, and In elements in the GaP:Zn/GaP/GaInP-GaP:Te/GaP samples and pure-GaP samples, respectively. The Avantage (v6.6.0) software was used to analyze the test results. The data were charge-corrected using the C 1s peak at 284.8 eV as a reference to compensate for binding energy shifts caused by charging effects, and peak fitting was performed for the high-resolution spectra of each element.

The C 1s orbital electron emission peak originates from the abundant OA ligands on the surface of the GaP nanoparticles. Since OA is a long-chain fatty acid with 18 carbon atoms, the C 1s peak is significantly higher than those of other elements. The Ga element in XPS exhibits characteristic peaks for the 2p_3/2_ and 2p_1/2_ orbital electrons at 1117.6 eV and 1144.4 eV, respectively, suggesting that the element Ga exists in the sample in the +3 oxidation state [41,42]. Oxidation is a common issue in the study of phosphide nanoparticles. During preparation, washing, and other processes, some of the P elements inevitably oxidize, forming PO_4_^3−^ defects on the GaP surface. Therefore, the P 2p XPS spectrum shows a GaPO_4_ peak at 133.2 eV (or 133.0 eV) and a GaP peak at 128.6 eV [42,43], as shown in Figure 4d. It is worth noting that the O 1s XPS spectrum also reflects the oxidation issues of the samples. Peak fitting analysis reveals two subpeaks at 531.0 eV and 533.2 eV (or 533.0 eV), where the former corresponds to the O atoms in the PO_4_^3−^ group, and the latter originates from the O atoms in the OA ligands on the surface of the GaP nanoparticles, which is consistent with reports in the literature [43,44]. Figure 5 clearly shows the elemental differences between GaP:Zn/GaP/GaInP-GaP:Te/GaP and pure GaP samples. In the pure GaP sample, the characteristic regions for Zn, In, and Te only exhibit irregular noise signals, with no corresponding elemental peaks detected, in line with the experimental expectations. On the other hand, the emission peaks in the characteristic regions for Zn, In, and Te in the GaP:Zn/GaP/GaInP-GaP:Te/GaP samples demonstrate the successful doping of three ions into the GaP nanoparticles through the thermal injection method. For example, the emission peaks at 1021.8 eV and 1044.9 eV originate from the 2p_3/1_ and 2p_1/2_ orbital electrons of Zn^2+^ [36], while the 3d_5/2_ and 3d_3/2_ orbital electrons of In^3+^ ions correspond to the 444.2 eV and 451.8 eV emission peaks [36,45]. Additionally, the XPS fine structure of Te shows peaks at 572.6 eV and 577.0 eV, corresponding to the Te 3d_5/2_ orbital and a peak indicating the presence of TeO_2_, mainly due to the oxidation of some Te [46].

### 3.4. Electroluminescence

**Figure 6:**
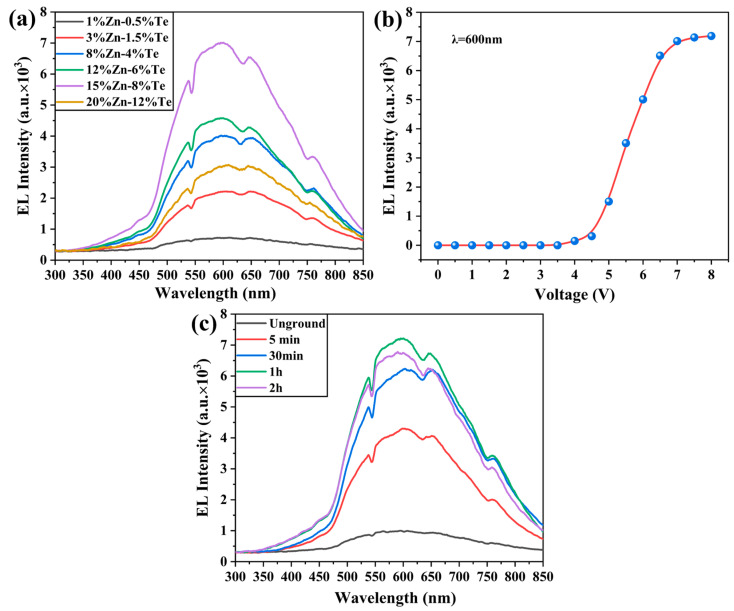
Electroluminescence of GaP:Zn/GaP and GaP:Te/GaP samples. (**a**) Different doping concentrations of Zn and Te. (**b**) Electroluminescence intensity of 15%Zn-8%Te sample recorded at 600 nm with different voltage excitation. (**c**) Effect of grinding time on the electroluminescence performance of 15%Zn-8%Te samples.

The electroluminescent properties of GaP nanoparticles are the primary focus of our research. Therefore, we tested the luminescence characteristics of annealed samples under electric field excitation using the classical indium tin oxide (ITO)–single insulating layer–aluminum electrode sandwich structure. As mentioned earlier, this research was inspired by gallium-based LEDs, and a series of experiments were conducted based on the GaP system. Modern GaP-LEDs typically grow an undoped GaP active layer and a GaInP quantum well layer outside the P/N-type layers to improve carrier injection efficiency and recombination luminescence efficiency. In order to facilitate rapid carrier recombination, the literature indicates that the hole concentration in LEDs is typically twice the electron concentration [47,48]. In summary, in the first stage of this study, Zn/Te doping was performed at a 2:1 ratio, and a single undoped GaP layer was coated to form GaP:Zn/GaP and GaP:Te/GaP core-shell nanoparticles. The ratio of the core-shell precursors and the reaction time of both nanoparticles was 1:1.4 and 1 h:3 h, respectively. The electroluminescent performance of the samples was tested using a USB2000 fiber optic spectrometer (Ocean Optics, Orlando, FL, USA) with an integration time of 100 ms, and the results are shown in Figure 6.

Figure 6a shows the electroluminescence spectra of GaP particles with different doping concentrations tested at a voltage of 7 V. It can be observed that, under electric field excitation, the GaP particles exhibit a broad emission peak ranging from 465 nm to 850 nm, with a central emission peak at 600 nm, as well as low-intensity emission in the 350 nm to 465 nm range. Meanwhile, the shape of the emission peak remains unchanged across samples with different doping concentrations. Based on related research results, we attribute this to three distinct electroluminescence pathways in GaP particles. Bulk GaP is an indirect bandgap semiconductor, with the highest point of its valence band and the lowest point of its conduction band located at the Γ point and X point in the Brillouin zone, respectively. The direct and indirect bandgaps correspond to 2.78 eV (446 nm) and 2.26 eV (548 nm), respectively [33]. Therefore, we infer that the electroluminescence peaks in the range of 350–465 nm originate from the low-efficiency intraband transitions of GaP, while the electroluminescence peak in the range of 465–543 nm arises from the band-edge electron transitions of GaP particles. Additionally, as previously mentioned, the GaP particles inevitably oxidized during preparation and washing, forming some GaPO_4_ defects on the particle surface. After high-temperature annealing, atomic rearrangement occurs, with some P atoms replaced by O atoms to form O_P_ defects, which combine with Zn_Ga_ defects to create Zn_Ga_-O_P_ complex centers. Zn and O occupy different lattice sites, forming various types of Zn_Ga_-O_P_ centers, such as nearest neighbors and second-nearest neighbors. The short-range complex centers exhibit higher energy and electron-hole capture efficiency [49]. Consequently, the carriers injected under the electric field are captured and recombine at various Zn_Ga_-O_P_ centers, producing the broad emission peak from 543 nm to 850 nm.

The doping concentration of Zn and Te influences hole and electron injection efficiency. As the doping concentration increases from 1%Zn-0.5%Te to 15%Zn-8%Te, the electroluminescence intensity of GaP particles increases steadily, reaching a maximum at 15%Zn-8%Te, where the relative intensity at 600 nm rises from 725.03 to 7012.39. However, at a doping concentration of 20%Zn-12%Te, the emission intensity decreases to 3061.89. Extensive reports in the literature indicate that excessive doping leads to lattice mismatch and the formation of defects, which act as nonradiative recombination centers, capturing and trapping carriers, thus reducing the luminescence intensity. Moreover, high doping concentrations reduce the distance between neighboring active ions, and the exciton energy is more likely to be released in a non-radiative form through inter-ion interactions, resulting in a decrease in luminescence efficiency. Additionally, excessive doping can also decrease carrier injection efficiency [50,51]. The synergistic effect of the above factors leads to an optimal doping concentration of 15%Zn-8%Te for GaP particles. We also investigated the optical response of the 15%Zn-8%Te sample to different voltages based on the emission intensity at 600 nm, as shown in Figure 6b. The results indicate that GaP particles first exhibit electroluminescence at 4 V, with the emission intensity increasing sharply as the voltage rises, peaking at 7 V with a corresponding working current of 140 mA. Beyond this point, the intensity growth stagnates, as the electron–hole injection–recombination rate inside the GaP particles reaches saturation. Further voltage increases do not significantly improve luminescence intensity. High working currents also cause significant sample heating, leading to carrier energy dissipation through phonon coupling, which results in thermal quenching and further reduces luminescence intensity [52]. Due to the severe agglomeration of GaP particles after annealing, we dispersed the particles through grinding and tested the electroluminescence performance of the 15%Zn-8%Te sample under different grinding durations, as shown in Figure 6c. The results reveal that samples ground for 1 h exhibit the best electroluminescence performance. Insufficient grinding results in oversized particles that hinder the formation of conductive circuits between the ITO and aluminum electrodes. Excessive grinding creates too many interparticle gaps, which hinders electron transport across the particles.

**Figure 7:**
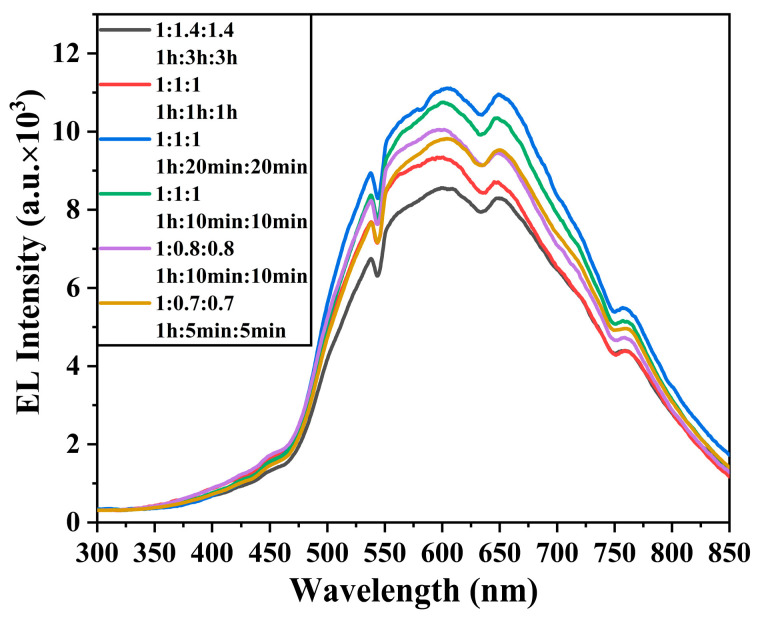
Electroluminescence of GaP:15%Zn/GaP/GaInP and GaP:8%Te/GaP samples with different core–shell precursor ratios and reaction times.

To further improve the electroluminescent performance of GaP particles, the second stage of this research coated a GaInP layer onto GaP:15%Zn/GaP particles and explored the effects of precursor ratios and reaction times on the luminescent performance of each layer structure of GaP:15%Zn/GaP/GaInP and GaP:8%Te/GaP. GaP can form a solid solution with In atoms at any concentration, where alloying reduces the bandgap of Ga_x_In_1-x_P, inducing a transition in the band structure from indirect to direct with increasing In concentration. Experimental and theoretical calculations show that the transition point occurs at approximately Ga_0.7_In_0.3_P [53,54]. Therefore, we choose Ga_0.91_In_0.09_P as the quantum well layer, which can firstly utilize its lower bandgap to effectively bind excitons and promote electron–hole complexation. Secondly, the indirect bandgap characteristics of Ga_0.91_In_0.09_P help maintain the consistency of the band structure in the GaP:15%Zn/GaP/GaInP system, avoiding the effects of indirect-to-direct bandgap transitions on carrier transport. Additionally, low-concentration In doping reduces lattice mismatch and strain at the interface, further optimizing the electroluminescent properties of GaP particles. Figure 7 presents the electroluminescent data of GaP:Zn/GaP/GaInP and GaP:Te/GaP samples tested at 7 V under various precursor ratios and reaction times.

The results indicate that the GaInP layer affects only the luminescence intensity of GaP particles, with no significant impact on the emission profile, suggesting that the introduction of GaInP does not alter the electroluminescence mechanism of the samples. It is worth noting that, in this research, GaP:Zn/GaP/GaInP and GaP:Te/GaP samples were prepared by using the thermal injection process of GaP QDs. Thus, the thickness of the functional layers in GaP nanoparticles is proportional to both the precursor ratio and reaction time [55,56]. As shown in Figure 7, the sample with a precursor ratio of 1:1:1 and a reaction time of 1 h:20 min:20 min exhibits the best electroluminescent performance, with an emission intensity of 11,109.21 at 600 nm, approximately 15 times that of the initial sample (with doping concentration, precursor ratio, and reaction time of 1%Zn-0.5%Te, 1:1.4, and 1 h:3 h, respectively). This enhancement is due to the fact that carriers cannot distribute uniformly across excessively thick GaP active layers and GaInP quantum well layers, resulting in insufficient exciton recombination in certain regions and a reduction in radiative emission efficiency. Reducing the thickness of functional layers can help mitigate this issue. However, when the thickness of the GaP active layer and GaInP quantum well layer becomes too thin, electrons and holes are not adequately confined, leading to partial exciton escape to other regions, which reduces the radiative recombination efficiency [57].

To further evaluate the research value of the samples, this study compared the electroluminescent performance of core–shell GaP particles with that of commercial AlGaInP-LED epitaxial wafers. Under the same spectral testing conditions, the relative luminous intensity of the AlGaInP-LED was 60,000. Although the AlGaInP-LED exhibited higher luminous intensity, it has drawbacks, such as complex preparation process, high production cost, high toxicity of raw materials, and limited applicability at micro and nano sizes. In contrast, this study used low-cost, low-toxicity raw materials to successfully prepare small-sized GaP particles through a simple thermal injection method, exhibiting excellent luminescent performance. In the future, further optimization of the structural model is expected to significantly improve the luminescent performance of GaP particles. In summary, through the structural design and parameter optimization of GaP particles, this study provides new ideas and methods for exploring high-performance miniature electroluminescent materials, demonstrating significant research value.

## 4. Conclusions

In this paper, doped GaP nanoparticles with core–shell structure were designed, multiple GaP:Zn/GaP/GaInP and GaP:Te/GaP samples were synthesized by using the thermal injection method, and the morphology, structure, and electroluminescence properties of the samples were systematically investigated. Experimental results demonstrated that these GaP nanoparticles, approximately 2–3 nm in size and with a classic zinc blende structure, are composed of seven elements: C, O, Ga, P, Zn, In, and Te. After high-temperature annealing, the GaP nanoparticles formed micron-scale aggregates, exhibiting significant electroluminescence under a 7 V external electric field through band-edge transitions and the Zn_Ga_-O_P_ recombination mechanism. Further studies revealed that the GaP:Zn/GaP/GaInP and GaP:Te/GaP samples, with doping concentrations, precursor ratios, and reaction times of 15%Zn-8%Te, 1:1:1, and 1 h:20 min:20 min, respectively, exhibited the best electroluminescent performance. Their relative luminescence intensity at 600 nm reached 11,109.21, approximately 15 times higher than that of the initial samples synthesized under conditions of 1%Zn-0.5%Te, 1:1.4, and 1 h:3 h. The experimental results confirmed that optimizing Zn and Te doping concentrations, as well as the thickness of the GaP and GaInP shell layers, effectively enhances carrier injection efficiency and radiative recombination efficiency under an electric field, thereby improving the electroluminescent performance of the samples. In conclusion, this research proposes a novel design approach for GaP-based electroluminescent materials, providing valuable insights for enhancing the performance of other types of luminescent materials.

## Figures and Tables

**Table 1:** List of experimental materials.

Materials	Chemical Formula or Abbreviation	Manufacturer	Purity
Gallium acetylacetonate	Ga(acac)_3_	Aladdin Biochemical Technology Co., Ltd., Shanghai, China	99.99%
Zinc acetylacetonate	Zn(acac)_3_	Aladdin Biochemical Technology Co., Ltd.	99.99%
Indium chloride	InCl_3_	Aladdin Biochemical Technology Co., Ltd.	99.99%
Tellurium powder	Te	Tianjin Damao Chemical Reagent Factory, Tianjin, China	99.99%
Oleic acid	OA	Tianjin Damao Chemical Reagent Factory	AR
1-Octadecene	ODE	Aladdin Biochemical Technology Co., Ltd.	GC
Tris(trimethylsilyl)phosphine	(TMS)_3_P	Macklin Biochemical Technology Co., Ltd., Shanghai, China	10 wt%
Trioctylphosphine	TOP	Aladdin Biochemical Technology Co., Ltd.	85%
Ethanol	CH_3_CH_2_OH	Guangdong Guanghua Technology Co., Ltd., Guangzhou, China	AR
N-hexane	C_6_H_14_	Sinopharm Chemical Reagent Co., Ltd., Shanghai, China	AR

## Data Availability

The data presented in this study are available on reasonable request from the corresponding author due to privacy.

## References

[1] Dupuis R.D., Krames M.R. (2008). History, development, and applications of high-brightness visible light-emitting diodes. J. Light. Technol..

[2] Han D.-P., Lee G.W. (2021). Comparative study of III-phosphide- and III-nitride-based light-emitting diodes: Understanding the factors limiting efficiency. Semicond. Sci. Technol..

[3] Piprek J. (2020). Efficiency models for GaN-based light-emitting diodes: Status and challenges. Materials.

[4] Usman M., Munsif M., Mushtaq U., Anwar A.-R., Muhammad N. (2020). Green gap in GaN-based light-emitting diodes: In perspective. Crit. Rev. Solid State Mater. Sci..

[5] Vanderwater D.A., Tan I.H., Hofler G.E., Defevere D.C., Kish F.A. (1997). High-brightness AlGaInP light emitting diodes. Proc. IEEE.

[6] Carreira J.F.C., Xie E., Bian R., Herrnsdorf J., Haas H., Gu E., Strain M.J., Dawson M.D. (2020). Gigabit per second visible light communication based on AlGaInP red micro-LED micro-transfer printed onto diamond and glass. Opt. Express.

[7] Li P., Zhang X., Qi L., Lau K.M. (2022). Full-color micro-display by heterogeneous integration of InGaN blue/green dual-wavelength and AlGaInP red LEDs. Opt. Express.

[8] Qi L., Li P., Zhang X., Wong K.M., Lau K.M. (2023). Monolithic full-color active-matrix micro-LED micro-display using InGaN/AlGaInP heterogeneous integration. Light Sci. Appl..

[9] Berg A., Yazdi S., Nowzari A., Storm K., Jain V., Vainorius N., Samuelson L., Wagner J.B., Borgstrom M.T. (2016). Radial Nanowire light-emitting diodes in the (Al_x_Ga_1-x_)_y_In_1-y_P material system. Nano Lett..

[10] Hai Q., Chen H., Sun C., Chen D., Qi Y., Shi M., Zhao X. (2023). Green-light GaN p-n junction luminescent particles enhance the superconducting properties of B(P)SCCO smart meta-superconductors (SMSCs). Nanomaterials.

[11] Qi Y., Chen D., Li Y., Sun C., Hai Q., Shi M., Chen H., Zhao X. (2024). Green-light p-n junction particle inhomogeneous phase enhancement of MgB_2_ smart meta-superconductors. J. Mater. Sci. Mater. Electron..

[12] Qi Y., Chen D., Sun C., Hai Q., Zhao X. (2024). The influence of electroluminescent inhomogeneous phase addition on enhancing MgB_2_ superconducting performance and magnetic flux pinning. Materials.

[13] Zhao X., Hai Q., Shi M., Chen H., Li Y., Qi Y. (2022). An improved smart meta-superconductor MgB_2_. Nanomaterials.

[14] Binks D.J., Dawson P., Oliver R.A., Wallis D.J. (2022). Cubic GaN and InGaN/GaN quantum wells. Appl. Phys. Rev..

[15] Ko M., Hong E., Eo Y.J., Lee S., Kim S., Kim H.J., Lee K.N., Kim C., Do Y.R. (2023). Development and isolation of dot LEDs for display applications through electrochemical etching and sonochemical separation. Adv. Funct. Mater..

[16] Park B., Lee J.K., Koch C.T., Wolz M., Geelhaar L., Oh S.H. (2022). High-resolution mapping of strain partitioning and relaxation in InGaN/GaN nanowire heterostructures. Adv. Sci..

[17] Liu N., Gocalinska A., Justice J., Gity F., Povey I., McCarthy B., Pemble M., Pelucchi E., Wei H., Silien C. (2016). Lithographically defined, room temperature low threshold subwavelength red-emitting hybrid plasmonic lasers. Nano Lett..

[18] Wang Y., Wang B., Sasangka W.A., Bao S., Zhang Y., Demir H.V., Michel J., Lee K.E.K., Yoon S.F., Fitzgerald E.A. (2018). High-performance AlGaInP light-emitting diodes integrated on silicon through a superior quality germanium-on-insulator. Photonics Res..

[19] Shenai-Khatkhate D.V., Goyette R.J., DiCarlo R.L., Dripps G. (2004). Environment, health and safety issues for sources used in MOVPE growth of compound semiconductors. J. Cryst. Growth.

[20] Oda O., Hori M. (2020). Novel epitaxy for nitride semiconductors using plasma technology. Phys. Status Solidi A.

[21] Peaker A.R. (1980). Light-emitting-diodes. Iee Proc. A Sci. Meas. Technol..

[22] Beppu T., Toyama M., Kasami A. (1972). GaP green light-emitting diodes with p-n-p-n structure. Jpn. J. Appl. Phys..

[23] Peaker A.R., Mottram A. (1972). Injection and quantum efficiencies of red-light-emitting gallium-phosphide diodes. J. Phys. D Appl. Phys..

[24] de Boer W.D., Timmerman D., Dohnalova K., Yassievich I.N., Zhang H., Buma W.J., Gregorkiewicz T. (2010). Red spectral shift and enhanced quantum efficiency in phonon-free photoluminescence from silicon nanocrystals. Nat. Nanotechnol..

[25] Miranda A., Serrano F.A., Vázquez-Medina R., Cruz-Irisson M. (2010). Hydrogen surface passivation of Si and Ge nanowires: A semiempirical approach. Int. J. Quantum Chem..

[26] Splendiani A., Sun L., Zhang Y., Li T., Kim J., Chim C.Y., Galli G., Wang F. (2010). Emerging photoluminescence in monolayer MoS_2_. Nano Lett..

[27] Lauth J., Strupeit T., Kornowski A., Weller H. (2012). A transmetalation route for colloidal gaas nanocrystals and additional III–V semiconductor materials. Chem. Mater..

[28] Gao S.M., Lu J., Zhao Y., Chen N., Xie Y. (2003). The growth process, stability of GaP nanocrystals and formation of Ga_3_P nanocrystals under solvothermal conditions in benzene. Eur. J. Inorg. Chem..

[29] Kher S.S., Wells R.L. (1994). A straightforward, new method for the synthesis of nanocrystalline GaAs and GaP. Chem. Mater..

[30] Kim Y.H., Jun Y.W., Jun B.H., Lee S.M., Cheon J.W. (2002). Sterically induced shape and crystalline phase control of GaP nanocrystals. J. Am. Chem. Soc..

[31] Macdougall J.E., Eckert H., Stucky G.D., Herron N., Wang Y., Moller K., Bein T., Cox D. (1989). Synthesis and characterization of III-V semiconductor clusters - GAP in zeolite-Y. J. Am. Chem. Soc..

[32] Micic O.I., Sprague J.R., Heben M., Lu Z., Curtis C.J., Nozik A.J. (1995). Synthesis and characterization of InP, GaP, and GaInP_2_ quantum dots. J. Phys. Chem..

[33] Kim S., Lee K., Kim S., Kwon O.P., Heo J.H., Im S.H., Jeong S., Lee D.C., Kim S.-W. (2015). Origin of photoluminescence from colloidal gallium phosphide nanocrystals synthesized via a hot-injection method. RSC Adv..

[34] Choi Y., Choi C., Bae J., Park J., Shin K. (2023). Synthesis of gallium phosphide quantum dots with high photoluminescence quantum yield and their application as color converters for LEDs. J. Ind. Eng. Chem..

[35] Almeida G., Ubbink R.F., Stam M., du Fossé I., Houtepen A.J. (2023). InP colloidal quantum dots for visible and near-infrared photonics. Nat. Rev. Mater..

[36] Gwak N., Shin S., Yoo H., Seo G.W., Kim S., Jang H., Lee M., Park T.H., Kim B.J., Lim J. (2024). Highly luminescent shell-less indium phosphide quantum dots enabled by atomistically tailored surface states. Adv. Mater..

[37] Won Y.H., Cho O., Kim T., Chung D.Y., Kim T., Chung H., Jang H., Lee J., Kim D., Jang E. (2019). Highly efficient and stable InP/ZnSe/ZnS quantum dot light-emitting diodes. Nature.

[38] Kim K., Suh Y.-H., Kim D., Choi Y., Bang E., Kim B.H., Park J. (2020). Zinc oxo clusters improve the optoelectronic properties on indium phosphide quantum dots. Chem. Mater..

[39] Pietra F., De Trizio L., Hoekstra A.W., Renaud N., Prato M., Grozema F.C., Baesjou P.J., Koole R., Manna L., Houtepen A.J. (2016). Tuning the lattice parameter of In_x_Zn_y_P for highly luminescent lattice-matched core/shell quantum dots. ACS Nano.

[40] Yin J.B., Zhao X.P. (2001). Temperature effect of rare earth-doped TiO_2_ electrorheological fluids. J. Phys. D Appl. Phys..

[41] Richards D., Zemlyanov D., Ivanisevic A. (2010). Assessment of the passivation capabilities of two different covalent chemical modifications on GaP(100). Langmuir.

[42] Yun S., Lee P.-C., Kuo C.-H., McLeod A.J., Zhang Z., Wang V., Huang J., Kashyap H., Winter C.H., Kummel A.C. (2024). Gallium phosphide conformal film growth on in-situ tri-TBP dry-cleaned InGaP/GaAs using atomic hydrogen ALD. Vacuum.

[43] Duan X., Ma J., Zhang W., Liu P., Liu H., Hao J., Wang K., Samuelson L., Sun X.W. (2023). Study of the interfacial oxidation of InP quantum dots synthesized from tris(dimethylamino)phosphine. ACS Appl. Mater. Interfaces.

[44] Virieux H., Le Troedec M., Cros-Gagneux A., Ojo W.S., Delpech F., Nayral C., Martinez H., Chaudret B. (2012). InP/ZnS nanocrystals: Coupling NMR and XPS for fine surface and interface description. J. Am. Chem. Soc..

[45] Kim K., Yoo D., Choi H., Tamang S., Ko J.H., Kim S., Kim Y.H., Jeong S. (2016). Halide-amine co-passivated indium phosphide colloidal quantum dots in tetrahedral shape. Angew. Chem. Int. Ed. Engl..

[46] Chen Q., Chen Y., Wang J., Liu M., Chen Z. (2022). The growth of high-quality hexagonal GaTe nanosheets induced by ZnO nanocrystals. Crystals.

[47] Lee H.J., Kim S.U., So S.J., Cho Y.D., Kim Y.J., Ahn S.C., Lee C.H. (2013). Reduction of surface defects on the GaP window layer of 630 nm AlGaInP LED using post-Zn diffusion process. Curr. Appl. Phys..

[48] Oh H.S., Ryu H.S., Park J.M., Lee H.J., Kim Y.J., Jang I.K., Park J.H., Kwak J.S., Baek J.H. (2014). Investigation of Mg doping profile in the p-cladding layer for high-brightness AlGaInP-based light emitting diodes. J. Nanosci. Nanotechnol..

[49] Feenstra R.M., McGill T.C. (1981). Defect reactions in GaP-(Zn,O). Phys. Rev. Lett..

[50] Hackett W.H., Rosenzwe W., Jayson J.S. (1969). Saturation of Zn-O complexes in GaP diodes. Proc. IEEE.

[51] Lin J.F., Wu M.C., Jou M.J., Chang C.M., Lee B.J. (1994). Effects of substrate misorientation and Zn doping characteristics on the performance of algainp visible light-emitting-diodes. Jpn. J. Appl. Phys. Part 2 Lett..

[52] Oh C.-H., Shim J.-I., Shin D.-S. (2019). Current- and temperature-dependent efficiency droops in InGaN-based blue and AlGaInP-based red light-emitting diodes. Jpn. J. Appl. Phys..

[53] Abdollahi A., Golzan M.M., Aghayar K. (2016). Electronic properties of Ga_x_In_1−x_P ternary alloy from first-principles. Comput. Mater. Sci..

[54] Degheidy A.R., Elwakil S.A.A., Elkenany E.B. (2013). Energy band structure calculations of Ga_x_In_1−x_P alloys under the influence of temperature and pressure. J. Alloys Compd..

[55] Chen H.S., Chen C.Y., Wu Y.C. (2024). High-performance giant InP quantum dots with stress-released morphological ZnSe-ZnSeS-ZnS Shell. Adv. Mater..

[56] Xie L., Harris D.K., Bawendi M.G., Jensen K.F. (2015). Effect of trace water on the growth of indium phosphide quantum dots. Chem. Mater..

[57] Oh H.S., Park J.M., Jung S.H., Lee D.W., Lee K.S., Kwon S.H., Park Y.T. (2019). Investigation of chirped well structures for broad-spectrum AlGaInP-Based light emitting diodes. J. Nanosci. Nanotechnol..

